# Synergy Effect of Nanocrystalline Cellulose for the Biosensing Detection of Glucose

**DOI:** 10.3390/s151024681

**Published:** 2015-09-24

**Authors:** Chakavak Esmaeili, Mahnaz M. Abdi, Aji P. Mathew, Mehdi Jonoobi, Kristiina Oksman, Majid Rezayi

**Affiliations:** 1 School of Chemical Sciences and Food Technology, Faculty of Science and Technology, Universiti Kebangsaan Malaysia, UKM, 43600 Bangi, Malaysia; E-Mail: chakavak486@yahoo.com; 2 Department of Chemistry, Faculty of Science, University Putra Malaysia, 43400 Serdang, Malaysia; 3 Institute of Tropical Forestry and Forest Products, University Putra Malaysia, 43400 Serdang, Malaysia; 4 Division of Materials Science, Composite Centre Sweden, Lulea University of Technology, 97187 Lulea, Sweden; E-Mails: aji.mathew@ltu.se (A.P.M.); kristiina.oksman@ltu.se (K.O.); 5 Department of Wood and Paper Science and Technology, Faculty of Natural Resources, University of Tehran, P.O. Box 31585-4313, 31587-77871 Karaj, Iran; E-Mail: mehdi.jonoobi@ut.ac.ir; 6 Chemistry Department, Faculty of Science, University Malaya, 50603 Kuala Lumpur, Malaysia; E-Mail: chem_rezayi@yahoo.com

**Keywords:** glucose biosensor, cellulose nanocrystals, GOx, PPy-CNC nanocomposite, chemical polymerization, direct electrochemistry

## Abstract

Integrating polypyrrole-cellulose nanocrystal-based composites with glucose oxidase (GOx) as a new sensing regime was investigated. Polypyrrole-cellulose nanocrystal (PPy-CNC)-based composite as a novel immobilization membrane with unique physicochemical properties was found to enhance biosensor performance. Field emission scanning electron microscopy (FESEM) images showed that fibers were nanosized and porous, which is appropriate for accommodating enzymes and increasing electron transfer kinetics. The voltammetric results showed that the native structure and biocatalytic activity of GOx immobilized on the PPy-CNC nanocomposite remained and exhibited a high sensitivity (*ca.* 0.73 μA·mM^−1^), with a high dynamic response ranging from 1.0 to 20 mM glucose. The modified glucose biosensor exhibits a limit of detection (LOD) of (50 ± 10) µM and also excludes interfering species, such as ascorbic acid, uric acid, and cholesterol, which makes this sensor suitable for glucose determination in real samples. This sensor displays an acceptable reproducibility and stability over time. The current response was maintained over 95% of the initial value after 17 days, and the current difference measurement obtained using different electrodes provided a relative standard deviation (RSD) of 4.47%.

## 1. Introduction

Cellulose is a renewable and biocompatible resource that displays unique mechanical, optical and electrical properties that make it suitable for materials applications, actuators/sensors, drug delivery systems, and biomedical science [1]. The nanofibers in natural cellulose consist of crystalline and amorphous domains that can be separated by acid hydrolysis [2]. Due to the high tensile strength, good water dispersibility, and hydrophilic properties of nanocellulose, it has been used to improve the mechanical and dispersibility properties of materials [1].

The electrostatic interactions between negatively charged nanocellulose and cationic species make them good candidates for application in sensors and permselective membranes to selectively transport species based on their charge. The permselective properties of nanocellulose thin films were studied by Thielemans [3]. Thielemans prepared a glassy carbon (GC) electrode modified with cellulose nanocrystals and showed that it can be used as a sensor and can selectively transport species based on their charge using cyclic voltammetry.

The nanostructure and high surface area of the supporting materials increase the surface loading of enzymes, enhancing the response time and performance of the sensor. In a sensor based on nanostructured materials, due to the porous structure and high surface area, penetration of the target molecules into the substrate is relatively faster, resulting in a higher sensitivity and faster response time [4]. Cellulose is the most abundant organic polymer found in nature that has been used as a biocompatible material for drug delivery [5] and has been shown to be suitable as a membrane for GOx stabilization [6]. Paper based materials show passive liquid transport, piezoelectricity, and biodegradability make them attractive low-cost functional materials for sensing devices [7].

Although cellulosic materials exhibit unique and advantageous properties, they also suffer from poor electrical conductivity. As such, cellulose does not exhibit an acceptable sensitivity for biosensor applications. In addition, cellulose is very hydrophilic [8], and this means that it is not compatible with some sensing molecules. Therefore, these biodegradable materials must be modified to use as supporting materials in biosensor applications.

Conducting polymers have frequently been used to modify nanocellulose to combine the electronic characteristics of the conjugated polymers with the high aspect ratio and porous structure of nanocellulose synergistically, resulting in excellent sensing mediators [9] Polyaniline (PANI) [10] and polypyrrole (PPy) [11] have been extensively used as electron-transfer pathways in GOx electrodes. Conducting polymers are capable of immobilizing enzyme antibodies or DNA and transduce the analytical signal generated by these biomolecules [12]. Conducting polymers are electroactive, and during the doping/dedoping process, ions are transferred inside the polymer structure. They have been used frequently in sensors, such as heavy metal sensors [13], hydrogen [14], acetic acid sensors [15] and microbial fuel cells [16].

These versatile materials have frequently been used in sensors and biosensors, but they suffer from low solubility and dispersibility in common organic solvents [17,18] as well as poor mechanical properties, which have led to their limited application in some electronic devices. Cellulosic materials with good water dispersibility and hydrophilic properties enhance the physical and structural properties of conducting polymers.

The ion exchange and mass transport capacity of conductive papers could also be improved by using nanomaterials, such as cellulose with a large specific surface area [19]. Over the last few decades, various analytical methods for determining trace amounts of glucose have been reported. These include surface plasmon resonance (SPR) [20], colorimetric [21], fluorescence [22], electrochemical [23], and other methods. Among these methods, the electrochemical glucose biosensor offers a high sensitivity, selectivity, simplicity and low cost of operation [24].

Enzymatic biosensors are the most studied type of biosensor in which a bio-recognition event is converted into a highly sensitive electrochemical signal [25]. Special attention is given to biosensors based on the combination of recognition reagent of glucose oxidize and electrochemical transducers [26]. Amperometric determination of H_2_O_2_ is the classical method of glucose detection in which a high anodic potential is needed to avoid possible interference from other biomolecules present in the system [27]. Nanomaterials can improve the electron transfer and eliminate or reduce the observed interference to detect H_2_O_2_ at a low potential [28,29].

Facile synthesis of tetragonal columnar-shaped TiO_2_ (TCS-TiO_2_) nanorods via electrochemical method was reported to be useful in rapid detection of glucose concentration in human serum [30]. The process of enzyme immobilization is a critical and important step to load the enzyme on the electrode while retaining the nature and biological activity of the enzyme without leaching during the analysis. Many approaches have been used for GOx immobilization, including covalent attachment [31], physical entrapment in a porous membrane [32], crosslinking [33] and so on.

Ternary gold nanoparticles/polypyrrole/reduced graphene oxide nanocomposite was used to facilitate GOx immobilization and a dynamic range of 0.2–1.2 mM (R^2^ = 0.986) with a sensitivity of 123.8 µA·mM^−1^·cm^−2^ was reported for this sensor [34]. An amperometric glucose biosensor was developed based on the physical adsorption of GOx in the Prussian Blue (PB)-modified screen-printed carbon electrodes (SPCEs) fiber matrix. This biosensor showed a linear calibration range between 0.25 mM and 2.00 mM (R^2^ = 0.987) and a detection limit of 0.01 mM glucose (S/N = 3) [35]. In another study, Rauf *et al*. used cellulose acetate-polymethylmethacrylate (CA-PMMA) membrane for immobilization of GOx [36]. They showed that the GOx retaining was 46% of the original activity as compared to the free enzyme at 20 °C.

One of the methods used to prevent the leaching of the GOx or the loss of the enzyme molecules from the electrode surface is to recover the electrode with triton X-100 as a surface active agent [37]. The analytical detection technique, immobilization method, as well as the activity and stability of the enzyme are important parameters that affect the biosensor performance [38].

There are different type of glucose meter in market named home blood glucose monitoring (HBGM) in lancing device or test strip which as quick and least painful blood glucose testing, have been proved to be overall easiest to use. However, HBGM showed lower accuracy compared to the laboratory glucose measurement and managers of laboratories believe that these immediate available devices are unacceptable for inpatient diabetes management. Therefore, the research on the preparation and fabrication new materials as sensing reagent and new techniques is still continuing in order to improve biosensors performance to bring them to the confidence of potential users and achieve standardization as commercial devices.

In this research paper, the advantages of the application of PPy/CNC nanocomposites in GOx adsorption and its electron transfer as a new sensing area for glucose detection is explored. Cellulose nanocrystal (CNC) was isolated from microcrystalline cellulose (MCC) using sulphuric acid hydrolysis, and pyrrole was chemically polymerized on the surface of individual cellulose nanocrystals to obtain nanocomposites with an electrically conducting continuous high surface area. The formed nanocomposite that retained the electronic characteristics of the conjugated polymers and the structural advantages of the cellulose nanocrystals was then used as a supporting material to prepare a modified electrode.

## 2. Experimental Section

### 2.1. Chemicals and Reagents

Glucose oxidase (GOx) (EC 1.1.3.4, 35.3 U·mg^−1^, Type II from Aspergillus niger, cholesterol and ascorbic acid (AA) all were obtained from Sigma-Aldrich (Dorset, UK). A colloidal suspension of cellulose nanocrystals (CNC) was prepared from microcrystalline cellulose (MCC) using acid hydrolysis using a protocol reported elsewhere [39]. Pyrrole was purchased from Merck (Stockholm, Sweden). Dipotassium hydrogen phosphate (K_2_HPO_4_), glucose, uric acid (UA) and Triton X-100 were purchased from Sigma-Aldrich (Dorset, UK); potassium dihydrogen phosphate (KH_2_PO_4_) was acquired from Merck (Darmstadt, Germany). All of the solutions were made with deionized water (D.I) throughout the study.

### 2.2. Instrumentation

All of the electrochemical experiments, including differential pulse voltammetry (DPV) and cyclic voltammetry (CV), were performed by an Auto Lab potentiostat with a three electrode electrochemical system consisting of a carbon pencil as the auxiliary electrode, Ag/AgCl as the reference electrode (3 M KCl), and a modified carbon screen printed electrode (SPE) as the working electrode. Field emission scanning electron microscopy images were recorded on a JEOL-JSM-7600F model (Hitachi, Tokyo, Japan), and the TEM images obtained by a LEO 912Ab (Hitachi, Tokyo, Japan) transmission electron microscope.

### 2.3. Synthesis of the PPy/CNC Nanocomposite

An aqueous stable colloidal suspension of cellulose nanocrystal (CNC) was used to prepare the PPy/CNC nanocomposite. The pyrrole monomer (pre-distilled), with a specific volume, was added into different concentrations of CNC ranging from 0.3 to 0.9 wt% with stirring at a medium speed of rotation using a magnetic stirrer. To initiate the polymerization, a solution containing an oxidizing agent (FeCl_3_) was added drop wise into the pyrrole and CNC mixture. The molar ratio of monomer to oxidizing agent was kept constant at 1:2. The polymerization process was carried out for 1 h at room temperature with moderate stirring before the black nanocomposite precipitate was filtered off and washed with distilled water several times by centrifugation. The nanocomposites were dried in an oven at 60 °C. The composite was kept in a desiccator before being characterized.

### 2.4. Preparation of Chemical Solutions

Typically, the PPy/CNC suspension was prepared by dispersing 1 mg of the PPy/CNC nanocomposite in 2 mL of deionized water and sonicated for 3 min until a dark homogeneous suspension solution was obtained. A phosphate buffered saline (PBS) solution was prepared by diluting a mixture of K_2_HPO_4_ (1.0 M) and KH_2_PO_4_ (1.0 M) stock solutions with an adjustment buffer solution (0.05 M, pH = 7.0). A GOx enzyme solution was prepared by dissolving 5 mg of the enzyme in 1 mL of PBS (0.05 M, pH = 7.0) that was stored at 4 °C when not in use. The glucose solution was freshly prepared from 1.0 × 10^−3^ to 2.0 × 10^−3^ M in PBS from stock solution.

### 2.5. Preparation of Modified Electrodes

Modified SPE/PPy/CNC/GOx electrodes were prepared based on a Layer-by-Layer (LbL) method: 10 µL of the PPy/CNC suspension was dropped on the SPE surface and allowed to dry at ambient temperature. It was found that when a small amount of the PPy/CNC suspension (e.g., 5 µL) was dropped onto the SPE surface, no significant signal was detected, and when a thick film of PPy/CNC was cast, it was not stable and leaked easily from the electrode surface after carrying out a few scanning cycles. Therefore, 10 µL of the suspension was used to obtain a good response and film stability. For immobilizing the enzyme, 20 µL of a fresh GOx solution was drop cast onto the PPy/CNC nanocomposite surface and then evaporated at room temperature. Because the sensor’s performance and sensitivity are affected by the enzyme concentration and activity, the SPE/PPy/CNC/GOx electrodes with varying concentrations of GOx ranging from 0.05 to 0.5 mg of the enzyme/electrode were prepared.

**Figure 1:**
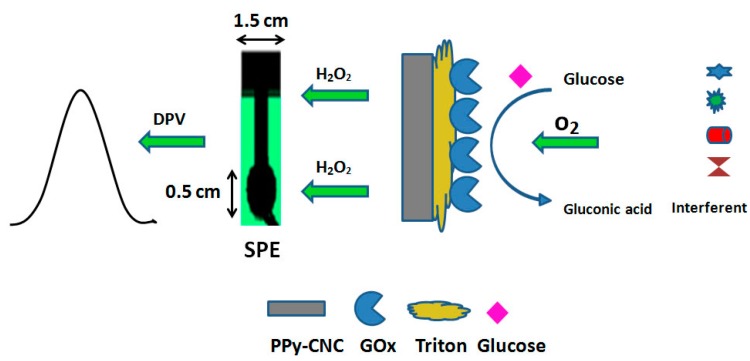
Schematic illustration of electrochemical glucose biosensor based on PPy/CNC as a membrane.

Finally, 5 µL of triton-x 100 (0.5 wt% in water) was used to prevent enzyme leaching from the surface of the electrode and was allowed to dry at 25 °C. Subsequently, the electrodes were washed with a PBS solution before any measurements to remove the unbound enzyme from the PPy/CNC surface. The electrode of SPE/PPy/GOx was prepared using the same method for comparison. The schematic view of LbL method for deposition of GOx on the PPy/CNC substrate is shown in Figure 1.

## 3. Results and Discussion

### 3.1. The Morphology of PPy and PPy/CNC Nanocomposites

**Figure 2:**
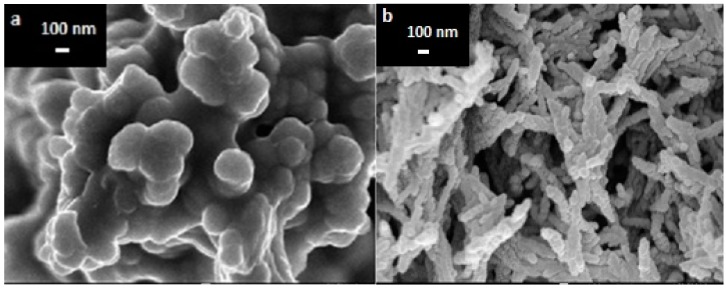
Microstructure of (**a**) PPy and (**b**) the porous PPy/CNC nanocomposite modified on the SPE surface.

The FESEM image of polypyrrole shown in Figure 2a shows a globular, dense and nonporous structure for PPy prepared from a 0.2 M pyrrole solution; the PPy/CNC formed an open and porous structure of intertwined fibers. From these images, no phase separation was detected, which indicates that the pyrrole polymerized homogenously on the CNC surface. The images of the PPy/CNC nanostructure showed a uniform size and fibrous and porous structure in the composite network via interconnection in a three-dimensional nanostructure. This is in agreement with previous observations [40]. The observed aggregates in the PPy/CNC membrane over the SPE surface appear to be regularly distributed (Figure 2b). The porous structure of the PPy/CNC nanocomposite provides a large surface area, with the GOx trapped inside the pores. The pores can accommodate a large quantity and allow the rapid diffusion of the active enzyme into the PPy/CNC membrane. As previously reported, the uniform and porous nanostructure of the substrate can considerably improve the effective surface of the electrode for loading biomolecules, resulting in an increase in the electron transfer kinetics [33].

### 3.2. Electrochemical Characterization

Voltammetric measurements were carried out on SPE/PPy/CNC/GOx and SPE/PPy/GOx nanocomposites, and the effects of the immobilized GOx and nanocomposite concentration, pH and buffer capacity, interference, and storage time on the current response of the prepared electrodes were studied. The capability of the electrochemical biosensor was also characterized in terms of its dynamic range, detection limit, reproducibility, and shelf life. All experiments were carried out at room temperature with a phosphate buffer (0.05 M, pH = 7) electrolyte solution under constant stirring conditions (100 rpm).

#### 3.2.1. Voltammetric Studies

**Figure 3:**
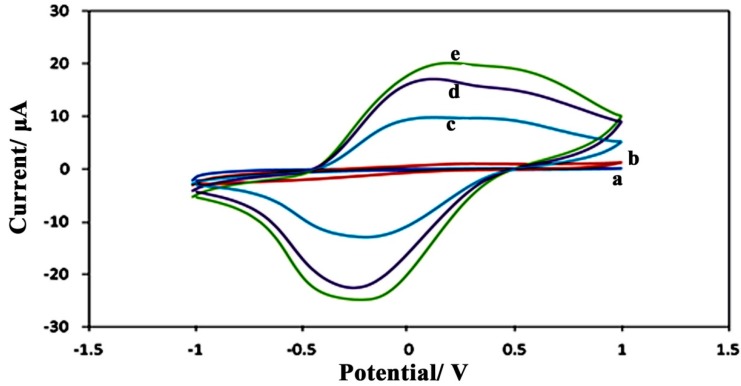
Cyclic voltammetry graphs of the unmodified bare SPE (a); the modified PPy/GOx electrode (b); and the PPy/CNC/GOx electrodes prepared using different concentrations of CNC of 0.3 (c); 0.5 (d); and 0.9 wt% (e).

Cyclic voltammetry (CV) of the various SPE/PPy/CNC and SPE/PPy electrodes was carried out in the scan range of −1.0 to +1.0 V, with a potential scan rate of 0.5 V·s^−1^ *versus* a Ag/AgCl electrode, and the results are shown in Figure 3. In bulky polypyrrole, electron transfer is relatively slow, and no obvious redox peaks were observed at the SPE/PPy electrodes from the voltammogram (Figure 3b). However, the SPE/PPy/CNC electrodes (Figure 3c–e) gave a couple of stable and well-defined redox peaks. In the presence of cellulose nanocrystals, the hydrogen-bonding interaction between the hydroxyl groups of cellulose and the hydrogen bonded to nitrogen on the PPy chain play a role, offering active sites for the formation of a polypyrrole chain on the surface of the cellulose nanocrystals. Cellulose nanocrystals (CNC) improve the active surface area of the bio-nanocomposite that could also be suitable for the immobilization of enzymes, enhance electron transfer, and increase the anodic current (Ip) in the voltammogram of the modified electrode prepared from a 0.3 wt% suspension of CNC. The CV of the SPE/PPy/CNC electrode is characterized by a pair of well-defined and almost symmetrical redox peaks with a cathodic (Epc) and anodic (Epa) peak potential of −0.24 and 0.29 mV, respectively (curve c). The formal potential (E^0^) of the SPE/PPy/CNC electrode prepared from a 0.3 wt% CNC suspension was estimated from the midpoint between the reduction and oxidation potentials to be 0.265 V.

The redox current decreased with an increase in the CNC concentration ranging from 0.3 to 0.9 wt%, which may be due to the agglomeration of the nanocrystals at higher concentrations, which is in agreement with the observed results by FESEM. The nanocomposite with a higher CNC content showed a larger size and lower porosity structure that resulted in a lower electron transfer and current peak by CV.

#### 3.2.2. Effect of the Immobilized GO_x_, Triton X-100, and PPy-CNC Concentration

**Figure 4:**
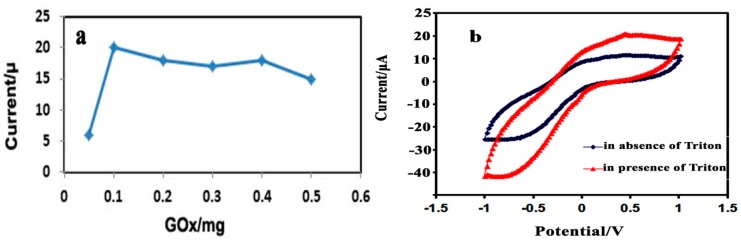
(**a**) Effect of the GOx concentration on the glucose biosensor response in PBS (0.05 M, pH = 7.0) at an applied potential of 0.36 V; (**b**) Effect of Triton X-100 on the glucose biosensor response.

During the stepwise modification of the electrode, the effect of the immobilized enzyme concentration on the glucose biosensor response was investigated. A varying concentration of GOx ranging from 0.05 to 0.5 mg/electrode was used to prepare the modified biosensor electrode. As can be observed from Figure 4a, an increased response was observed from 0.05 to 0.1 mg of GOx, and the electrode prepared using 0.1 mg of GOx for catalytic glucose oxidation showed a high and well-defined current response, indicating that the concentration was sufficient to cover the PPy-CNC composite surface to consume O_2_ and produce H_2_O_2_ [41]. In this region, glucose oxidation is kinetically limited, where the oxygen and glucose consumption is directly proportional to the GOx concentration. With a further increase in the amount of enzyme, no considerable change is observed in the current intensity because the reaction is diffusion limited, which means that the reaction kinetics are governed by the mass transport of the analyte from the bulk into the sensor.

At higher enzyme concentrations, the buffering capacity of the sensor controls the performance, resulting in a local decrease in pH that reduces the activity of the enzyme. Another possible reason for the activity reduction may be that at a high concentration of GOx, glucose is consumed rapidly at the outer surface of the sensor, which is not proportional to the decreasing oxygen level, resulting in a loss of sensitivity [42]. The results are consistent with previous literature reports [23,43].

The effect of Triton on the SPE modified electrode performance was studied using 0.5% Triton X-100 solution. As it can be seen from Figure 4b, the modified electrode covered with triton showed higher current response compared to the modified electrode without triton at 0.36 V indicating Triton X-100 being able to prevent GOx leaching from the surface of electrode.

The PPy-CNC nanocomposite is highly hydrophobic and strongly adsorbed onto the carbon SPE surface. It provided a high surface area and a porous structure that is suitable for the attachment of the GOx enzyme. This facilitates a direct electron transfer at a higher rate between the GOx biomolecule and the electrode surface during the enzymatic reaction [11]. Furthermore, the DPV response of the biosensor increased with an increasing nanocomposite concentration until 0.005 mg/electrode was reached. This observation is in agreement with the maximum amount of nanocomposite for enzyme loading within the nanostructure [44] and allowed the measurement of DPV in the presence of various concentrations of glucose to be carried out.

#### 3.2.3. Effect of pH and Buffer Capacity

**Figure 5:**
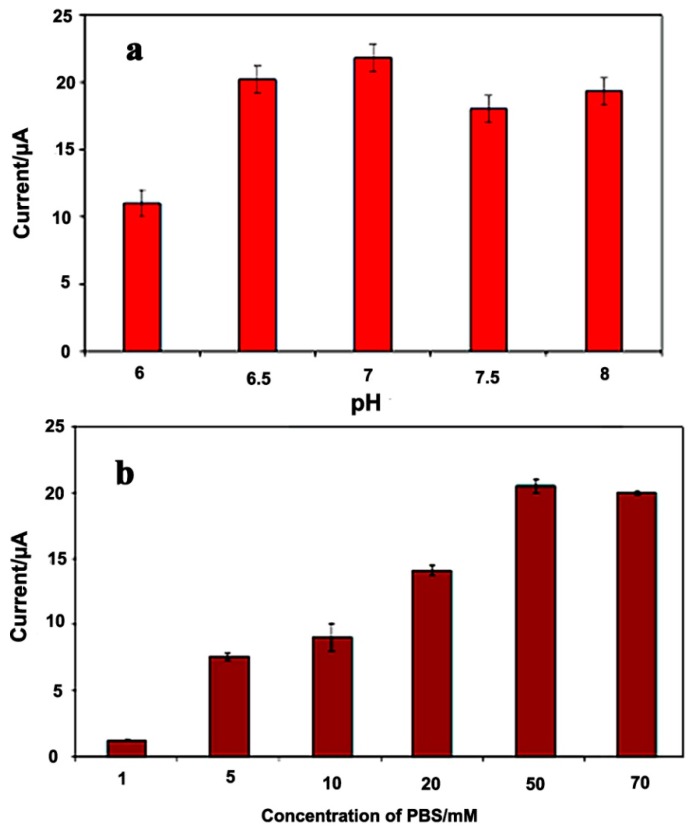
Effect of the pH (**a**) and ionic strength (**b**) of PBS on the PPy/CNC/GOx based glucose biosensor response in a 10 mM glucose solution at an applied potential of 0.36 V.

To enhance the electrocatalytic activity of the sensor toward glucose, the modified electrodes were optimized with PBS solution with different capacities ranging from 1.0 to 70 mM and different pHs within the range from 6.0 to 8.0. The pH of the solution effects the GOx activity [45]. From Figure 5a, it can be observed that the maximum response is approached at a pH of 7.0. Because the isoelectric point of GOx is 4.2, it carries a net negative charge at pH = 7 [46]. The electrostatic interactions between the negatively charged enzyme and the positively charged polypyrrole matrix at a pH of 7, results in an increased adsorption and incorporation of the enzyme into the nanostructure, resulting in a higher sensitivity at this pH.

This enzyme is more active in neutral solutions and maintains its natural structure at this pH [47]. Under acidic and more basic conditions, the immobilization of GOx onto the electrode surface is minimized. On the other hand, under acidic conditions, decreased enzyme activity may arise because the enzyme is denatured [30]. Furthermore, a pH of 7.0 provides a suitable environment where the interactions between the redox ions and the electrode surface are optimal and hence results in the superior response observed when the pH of the enzymatic reaction is selected to be 7.0.

The sensor response strongly depends on the concentration of PBS in the solution, as shown in Figure 5b. The current intensity was found to increase 20-fold when the PBS concentration changed from 1.0 to 50 mM. This may be because the role of the PBS solution is to transport protons between the GOx and the PPy-CNC membrane, which is called the “carrier-mediated” process [48]. The effect of the buffer concentration on the glucose biosensor intensity is restricted owing to the O_2_ limitation [49].

#### 3.2.4. Electrochemical Response Studies of the Glucose Biosensor

**Figure 6:**
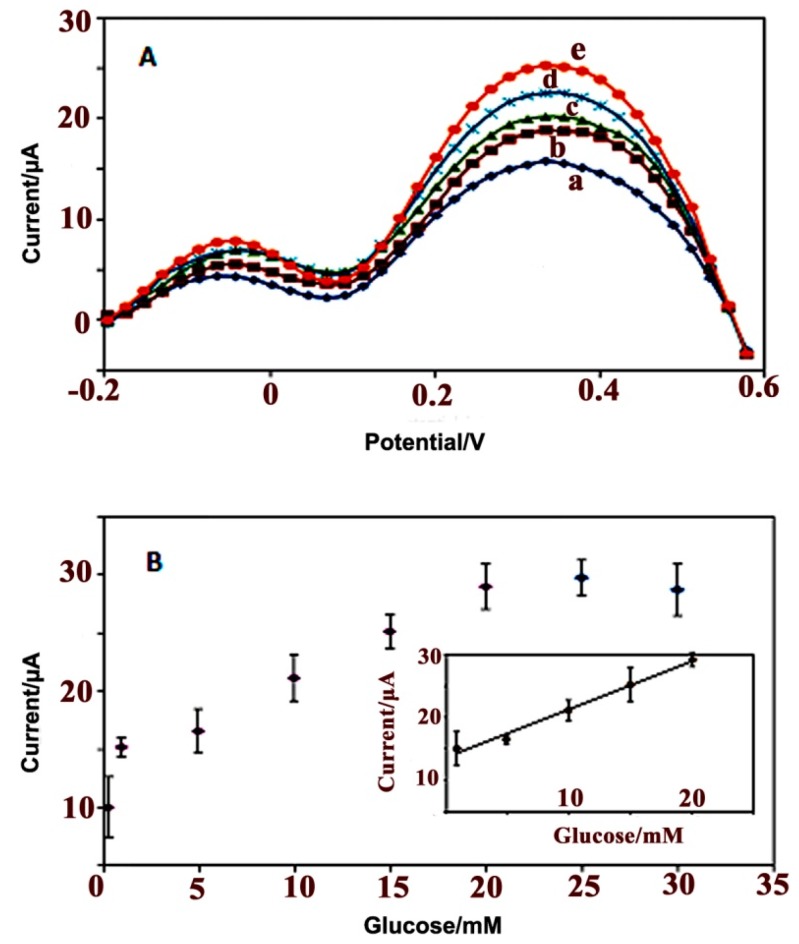
DPV graph (**A**) obtained using various glucose concentrations of (a) 1, (b) 5, (c) 10, (d) 15, and (e) 20 mM; and the analytical curve (**B**) of the PPy/CNC/GOx based glucose biosensor in a buffer solution (50 mM, pH = 7) at a scan rate of 0.5 V/s *versus* a Ag/AgCl electrode.

Figure 6A shows the DPV plot and analytical curves for the PPy/CNC/GOx modified electrode on successive additions of glucose at a scan rate of 0.5 V/s *versus* the Ag/AgCl electrode in 50 mM PBS with a pH of 7.0. The dynamic response range of the glucose biosensor was determined by using difference glucose concentrations, ranging from 0.3 to 30 mM. It should be noted that the enzymatic biosensor showed good linearity in the detection of glucose within the concentration range of 1–20 mM (Figure 6B, inset) with a sensitivity of 0.73 μA·mM^−1^ and regression coefficient (R^2^) of 0.989.

According to Michaelis-Menten’s model, by increasing analyte concentration, the rate of catalytic reaction increases linearly to reach a maximum at higher substrate concentrations in which all of the available enzyme has been converted to enzyme substrate complex.

In our research, the increasing of glucose concentration from 1.0 to 20 mM caused enzymatic reaction to proceed faster resulting higher concentration of hydrogen peroxide and higher current response. The maximum rate achieved at 20 mM and then began to level off at higher concentrations. This possibly is due to this fact that as the reaction proceeds (Equation (1)) the concentration of gluconic acid increases lead to decreasing the pH of immediate environment on the surface of electrode. The decreasing of pH would alter or totally inhibit the enzyme from catalyzing reaction. Another reason is that the sensitivity of the biosensor is affected by the dissolved oxygen concentration. The increasing glucose concentration after 20 mM will lead to increase consumption of oxygen and decreases sensitivity of electrode.

The limit of detection (LOD) was defined as the cross-section of both extrapolated linear portions of the calibration curve statistical analytical method and was calculated to be (50 ± 10) µM. Due to the electrocatalytic activity of the GOx enzyme (Equation (1)).

(1) $$\text{Glucose}+{\text{O}}_{2}\overset{\text{Gox}}{\to }\text{Gluconic acid }+{\text{H}}_{2}{\text{O}}_{2}$$

The O_2_ consumption and the H_2_O_2_ production increased during the reaction upon successive additions of glucose. No significant signals were observed for the sample tested in the absence of glucose [50]. Increasing the glucose concentration by more than 20 mM showed no effect on the biosensor response.

#### 3.2.5. Reproducibility, Stability and Interference Studies

The reproducibility of the glucose biosensor was estimated by measuring the current response of five different modified electrodes with the same glucose solution (10 mM glucose) and under the same conditions within one day. The current difference measurement obtained using the different electrodes provided a RSD of 4.47%.

**Figure 7:**
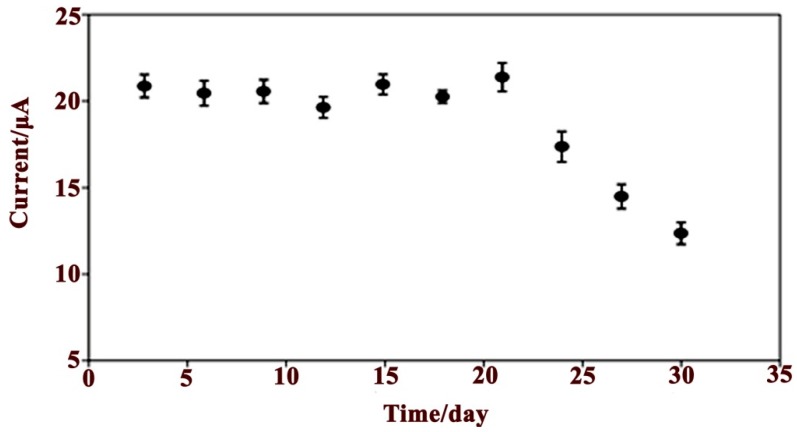
The life time of the PPy/CNC/GOx modified electrode based on the DPV response for a 10 mM glucose solution in PBS (0.05 M, pH = 7).

The shelf life and storage stability of the glucose biosensor was tested during a period of one month in the presence of a 10 mM glucose solution with PBS (0.05 M, pH 7.0) and is shown in Figure 7.

The electrode was stored at 4 °C under dry conditions after each measurement. After three days, no significant current differences were observed, but the biosensor sensitivity decreased slowly, reaching 95% of the initial sensitivity at day 17. Then, the sensitivity decreased rapidly and an activity loss of 80.57% was observed after four weeks, reaching 19.4% of the initial sensitivity at day 30.

The good stability of the sensor may be attributed to the biocompatibility of the PPy/CNC nanostructure, which provides a promising microenvironment for GOx to retain its bioactivity. Another possible reason may be the high surface area and porosity of the modified electrode that enhances the immobilization of the GOx enzyme molecules. After four weeks, the stability decreased because of the reduced enzyme catalytic activity and conductivity of the PPy/CNC nanocomposite. The PPy/CNC-based glucose biosensor compared with other glucose biosensors reported in the literature showed better stability. For example, the stability of the GOx immobilized on the PPy/carbon nanotube composite matrix decreased over 10 h [51], and modified Au electrodes based on silver nanotubes lose their enzyme catalytic activity after two weeks [52].

**Figure 8:**
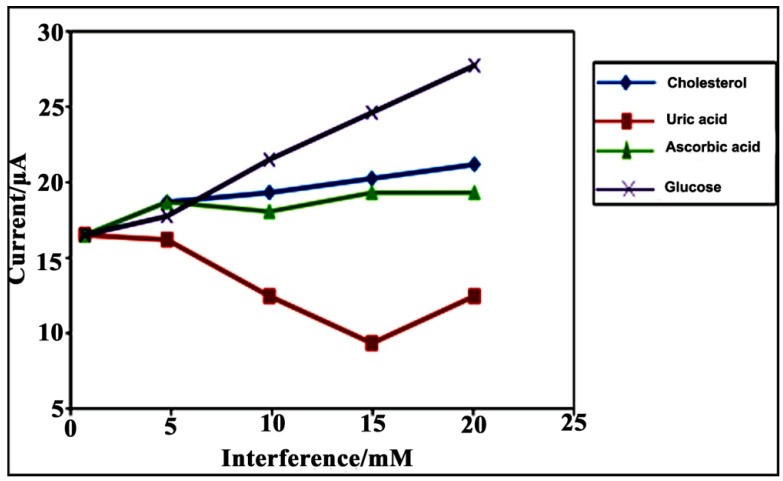
The effect of interfering species on the glucose biosensor in the presence of PBS (0.05 M, pH = 7.0).

The separate solution method (SSM) has been used to investigate the effect of interference on the glucose biosensor response. The measurement of interference based on the SSM method was performed in a glucose solution and interfering species, including cholesterol, uric acid (UA) and ascorbic acid (AA), separately in PBS (50 mM, pH = 7.0). The concentration of the interfering species utilized the boundaries of the linear response range from 1.0 × 10^−3^ to 2.0 × 10^−2^ M [53]. As shown in Figure 8, after the addition of interfering species, there was no obvious increase in the current response of the glucose biosensor.

These results show that the glucose biosensor based on PPy/CNC as a conducting membrane has the ability to perform as a glucose sensor with no interference. The glucose biosensor utilizing PPy/CNC as the immobilization membrane developed in this study appeared to improve the linear range compared to other types of sensors using a different matrix. In Table 1, the glucose biosensor using the PPy/CNC/GOx modified electrode is compared favorably with other glucose biosensors in terms of the dynamic range response, stability, and limit of detection (LOD).

**Table 1:** Comparison of PPy/CNC/GOx-based electrochemical glucose biosensors with previously reported literature biosensors.

Electrode	Detection Methods	Linear Range (mM)	LOD (mM)	Long-Term Stability (Days)	References
Py/CNC	DPV	1–20	0.05	21	Present work
Graphene/CdS nanocrystals	CV and impedance spectroscopy	2–16	0.7	-	[54]
Hydrophilic cellulose paper	Amperometric	1–5	0.18	110	[55]
Graphene/AuNPs/Chitosan nanocomposites	CV	2–14	0.18	15	[41]
silica sol–gel	Fluorescence	0.1–5	0.06	30	[56]
ZnO nanorods	Amperometric	0.01–3.45	0.01	7	[57]
Colloidal gold modified carbon paste electrode	CV	0.04–0.28	0.01	-	[58]
PPy/CNT	Amperometric	0–50	0.2	75 min	[59]

## 4. Conclusions

Here, a novel PPy-CNC bionanocomposite was synthesized by a chemical polymerization method. Due to the synergistic effect between CNC and PPy, a good electrochemical behavior and the electron transfer between the GOx and the modified electrode was observed. In summary, nanocomposites can provide unique properties to design glucose biosensors using a simple method based on the entrapment of the GOx on the membrane surface. Polymerization of pyrrole on the nanocellulose could enhance surface area and porosity structure of nanocomposite resulting good electrocatalytic activity toward biomolecules. The porous structure formed in the presence of nanocellulose provided strong adsorption ability for the immobilization of GOx, In addition crystalline structure of nanocellulose could facilitate electron transfer between electrode and GOx with strong catalytic properties.The electrochemical and DPV responses of the GOx for glucose biosensor detection were examined in detail. The large surface to volume ratio of the PPy/CNC nanocomposite modified SPE is sufficient to impact the current response without the addition of any crosslinking agents or modifiers. Therefore, this membrane established a novel sensing area for the detection of glucose and provided a general route to fabricate a glucose biosensor based on the immobilization of GOx onto a PPy/CNC membrane. This sensor, with a wide dynamic range, good stability, reproducibility, high sensitivity and fast electron transfer, can be applied in a broad range of fields potentially including biomedical engineering, chemical engineering, medical analysis and biochemistry and may also be a good candidate for the immobilization of biomolecules and the fabrication of third-generation biosensors.
